# Supplementary material for: Association between self-esteem and suicide risk in adolescents from five schools in northern Peru: A cross-sectional study
Source: Glob Ment Health (Camb). 2026 Feb 20;13:e68. doi: 10.1017/gmh.2026.10155 (PMC13112299; doi:10.1017/gmh.2026.10155)
Supplement: Valladares-Garrido et al. supplementary material [file S2054425126101551sup001.zip › S2054425126101551sup001/Supplementary material file 1.pdf]

USMP
UNIVERSIDAD DE SAN MARTÍN DE PORRES
Facultad de Medicina Humana

La Molina, 21 de marzo de 2023

Oficio No. 348 - 2023 - CIEI-FMH- USMP

Señorita
Jassmin del Milagro Santin Vasquez
Alumna de pregrado
Facultad de Medicina Humana
Universidad de San Martín de Porres
Presente

Ref. Plan de Tesis: Asociación entre acné y trastornos de salud mental en adolescentes del nivel secundario de Lambayeque, 2021.

De mi consideración:

Es grato expresarle mi cordial saludo y en atención a la solicitud de **Luz Angelica Aguilar Manay y Jassmin del Milagro Santin Vasquez**, alumnas de pregrado informarles que, en cumplimiento de las buenas prácticas clínicas y la legislación peruana vigente en materia de investigación científica en el campo de la salud, el Comité de mi presidencia, **sesión del 20 de marzo evaluó y aprobó el siguiente documento:**

- **Plan de Tesis: Asociación entre acné y trastornos de salud mental en adolescentes del nivel secundario de Lambayeque, 2021.**

Es cuanto informo a usted para su conocimiento y fines que correspondan.

Atentamente,

USMP
UNIVERSIDAD DE SAN MARTÍN DE PORRES

Dr. Amador Vargas Guerra
Presidente
Comité Institucional de Ética en Investigación de la Facultad de Medicina Humana de la Universidad de San Martín de Porres

AVG/ABZ/ach

PD. El presente formato (hoja membretada) será utilizado a partir del 07 de febrero de 2023 para las comunicaciones del CIEI, debido al cambio del logo de la Universidad de San Martín de Porres.

Comité Institucional de Ética en Investigación
Facultad de Medicina Humana USMP
Alameda del Corregidor N° 1531 Urb. Los Sirius
III Etapa – La Molina
Telf:365-2300 anexo 160
etica_fmh@usmp.pe
https://medicina.usmp.edu.pe/comite-etica-usmp-fmh/
