# Supplementary material for: Association between self-esteem and suicide risk in adolescents from five schools in northern Peru: A cross-sectional study
Source: Glob Ment Health (Camb). 2026 Feb 20;13:e68. doi: 10.1017/gmh.2026.10155 (PMC13112299; doi:10.1017/gmh.2026.10155)
Supplement: Valladares-Garrido et al. supplementary material [file S2054425126101551sup001.zip › S2054425126101551sup001/Supplementary material file 2.pdf]

Asentimiento Informado

"Asociación entre acné y trastornos de salud mental en adolescentes del nivel secundario de Lambayeque, 2021"

1) Instituciones : Universidad San Martin de Porres
Investigador principal : Aguilar Manay, Luz Angélica; Santin Vasquez Jassmin del Milagro.
Título : Asociación entre acné y trastornos de salud mental en adolescentes del nivel secundario de Lambayeque, 2021"
Propósito del Estudio:
Estamos invitando a usted a participar en un estudio llamado: "Asociación entre acné y trastornos de salud mental en adolescentes del nivel secundario de lambayeque, 2021". Este es un estudio desarrollado por las investigadoras de la Universidad San Martin de Porres: Aguilar Manay, Luz Angélica; Santin Vasquez Jassmin del Milagro. El propósito de este estudio es comprender la influencia de la presencia de acné en la salud mental de adolescentes de Lambayeque
Su ejecución ayudará a conocer e identificar la prevalencia de depresión, ansiedad y estrés y su asociación con la presencia de acné y otros factores socioeducativos.
Procedimientos:
Si Usted decide participar en este estudio se le realizará lo siguiente:
- Se le encuestará con algunas preguntas para marcar de opción múltiple.
La encuesta puede demorar unos 15 minutos. Los resultados de las encuestas se almacenarán respetando la confidencialidad y el anonimato.
Riesgos:
Su participación en el estudio no supone ningún riesgo para su persona.
Beneficios:
Usted se beneficiará al conocer acerca de la potencial asociación de los trastornos de salud mental y presencia de acné en los adolescentes del nivel secundario de la región Lambayeque.
Costos e incentivos
Usted no deberá pagar nada por la participación. Igualmente, no recibirá ningún incentivo económico ni medicamentos a cambio de su participación.
Confidencialidad:
Nosotros guardaremos la información con códigos y no con nombres. Si los resultados de este estudio son publicados, no se mostrará ninguna información que permita la identificación de Usted. Sus archivos no serán mostrados a ninguna persona ajena al estudio.
Derechos del participante:
Si usted se siente incómodo durante la encuesta podrá retirarse de éste en cualquier momento, o no participar en una parte del estudio sin perjuicio alguno. Si tiene alguna inquietud y/o molestia, no dude en preguntar al personal del estudio. Puede comunicarse con Aguilar Manay, Luz Angélica; Santin Vasquez Jassmin del Milagro, investigadoras principales del estudio (correo: luz_aguilar2@usmp.pe) y/o al Comité que validó el presente estudio, Dr. Amador Vargas Guerra, Presidente del Comité de Ética para la investigación de la Universidad San Martin de Porres, correo: etica_fmh@usmp.pe
ASENTIMIENTO
Acepto voluntariamente participar en este estudio, comprendo que cosas pueden pasar si participo en el proyecto, también entiendo que puedo decidir no participar, aunque yo haya aceptado y que puedo retirarme del estudio en cualquier momento:

◯ No deseo participar en el estudio
◯ Si deseo participar en el estudio
