# Supplementary material for: Association between self-esteem and suicide risk in adolescents from five schools in northern Peru: A cross-sectional study
Source: Glob Ment Health (Camb). 2026 Feb 20;13:e68. doi: 10.1017/gmh.2026.10155 (PMC13112299; doi:10.1017/gmh.2026.10155)
Supplement: Valladares-Garrido et al. supplementary material [file S2054425126101551sup001.zip › S2054425126101551sup001/Supplementary material file 3.pdf]

Autorización de padres de familia

"Asociación entre acné y trastornos de salud mental en adolescentes del nivel secundario de Lambayeque, 2021"

1) Record ID ______________________________

2) AUTORIZACIÓN DE PADRES DE FAMILIA PARA PARTICIPACIÓN DE MENORES DE EDAD EN ENCUESTA VIRTUAL

Estimado padre, madre o tutor:
Se le está pidiendo a su menor hijo que forme parte una encuesta virtual conducida por Jassmin Santin Vasquez y Luz Angelica Aguilar Manay, estudiantes de la Facultad de Medicina Humana de la Universidad San Martín de Porres.
La presente investigación se titula "Asociación entre acné y trastornos de salud mental en adolescentes del nivel secundario de Lambayeque, 2021"

Esta es una encuesta virtual de suma importancia ya que tiene como propósito conocer si en los escolares del nivel secundario existe asociación entre acné y trastornos de salud mental en adolescentes.

La participación de su menor hijo es voluntaria. La encuesta dura 15 minutos de su tiempo.
La información obtenida en esta encuesta virtual será únicamente utilizada para la elaboración de una tesis. La identidad de su menor hijo(a) será tratada de manera anónima, es decir, el investigador no conocerá la identidad de quién completó la encuesta.

Asimismo, su información será analizada de manera conjunta con la respuesta de sus compañeros y servirá para la elaboración de artículos y presentaciones académicas.

Si está de acuerdo con los puntos anteriores, complete sus datos a continuación:

- ◯ No doy mi autorización para que mi hijo complete la encuesta
- ◯ Doy mi autorización para que mi hijo complete la encuesta
